# A unique case of bilateral nanophthalmos and pigmentary retinal abnormality with unilateral angle closure glaucoma and optic disc pit

**DOI:** 10.1186/s12886-023-03132-8

**Published:** 2023-09-26

**Authors:** Prerna Garg, Brajesh Kumar, Suneeta Dubey

**Affiliations:** https://ror.org/03fwpw829 grid.440313.1 0000 0004 1804 356X Dr. Shroff Charity Eye Hospital, Kedarnath Lane, 110002 New Delhi, India

**Keywords:** Nanophthalmos, Optic disc pit, Angle-closure disease, Optic disc drusen

## Abstract

**Background:**

Microophthalmos or ‘dwarf eye’ is characterized by an axial length 2 standard deviation less than age-matched controls. It is classified into nanophthalmos, relative anterior microphthalmos, and posterior microphthalmos based on the anterior segment: posterior segment ratio. Nanophthalmos can occur in association with optic disc drusen, foveoschisis, and retinitis pigmentosa, as an autosomal recessive syndrome linked to mutations in the MFRP gene. We report a case of bilateral nanophthalmos and pigmentary retinopathy with angle closure glaucoma and optic disc pit in one eye. We believe this to be the first case presenting with optic disc pit in association with nanophthalmos.

**Case presentation:**

A 56-year-old female presented with bilateral small eyes, high hypermetropia, shallow anterior chamber depth, increased lens thickness, mid-peripheral retinal flecks, and macular edema. She also had high intraocular pressure in the right eye, with a disc cupping of 0.9 with an Optic disc pit. The macular edema in the right eye was found to occur in association with the Optic disc pit, whereas, in the left eye, it was associated with intra-retinal hemorrhages and diagnosed as macular branch retinal vein occlusion secondary to hypertension. She was started on anti-glaucoma medications in both eyes and planned for Anti-VEGF injection in the left eye.

**Conclusion:**

This case report is unique as it reports an association of Nanophthalmos with Optic Disc pit, with an associated angle closure glaucoma in the same eye, an association which has never been previously reported in the literature.

## Background

Nanophthalmos (NO), or total microphthalmos, was first described by Warburg as a developmental arrest of ocular growth. [1] It is characterized by an axial length (AL) 2 standard deviation (SD) less than age-matched controls, with microcornea, increased corneal curvature, shallow anterior chamber depth (ACD), increased lens/eye volume ratio, narrow iridocorneal angles, high hyperopia (usually > + 8D) and thickened sclera. [2]

Fundus in these patients may be normal with only a crowded disc or may show various pathologies like optic disc drusen, macular/ foveal hypoplasia, foveal schisis/cysts, mid-peripheral retinal flecks/ retinitis pigmentosa like picture. [3] Choroidal effusions and exudative retinal detachments occur more commonly due to the thickened sclera obstructing the vortex vein outflow pathway. [4]

NO has been noted to occur as a part of an ocular syndrome when associated with Foveoschisis, Optic disc Drusen, and Retinitis Pigmentosa-like findings. It is an autosomal recessive syndrome found to be linked to mutations in the MFRP gene. [5]

We report a case of bilateral NO in a female associated with pigmentary retinal changes with the unique finding of an optic disc pit in one eye, coincidentally associated with angle closure glaucoma also in the same eye.

## Case report

**Fig. 1:**
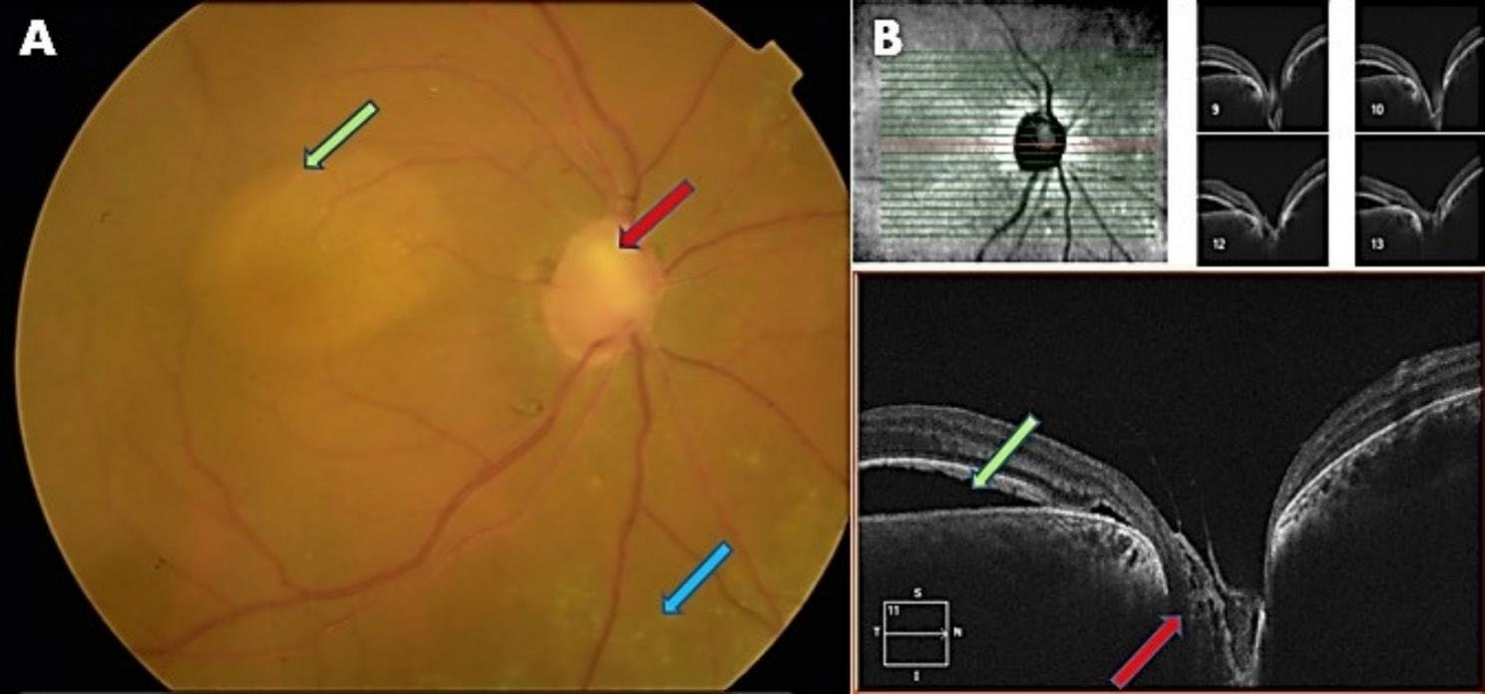
Color Fundus photo and OCT of the Right eye. **A**. Fundus picture showing advanced glaucomatous damage with an Optic disc pit (Red arrow) in the superotemporal quadrant, maculopathy (Green arrow), and yellowish flecks (Blue arrow) in the mid-periphery. **B**. OCT image depicting an Optic disc pit (Red arrow) associated with subretinal fluid in the macular region (Green arrow)

**Fig. 2:**
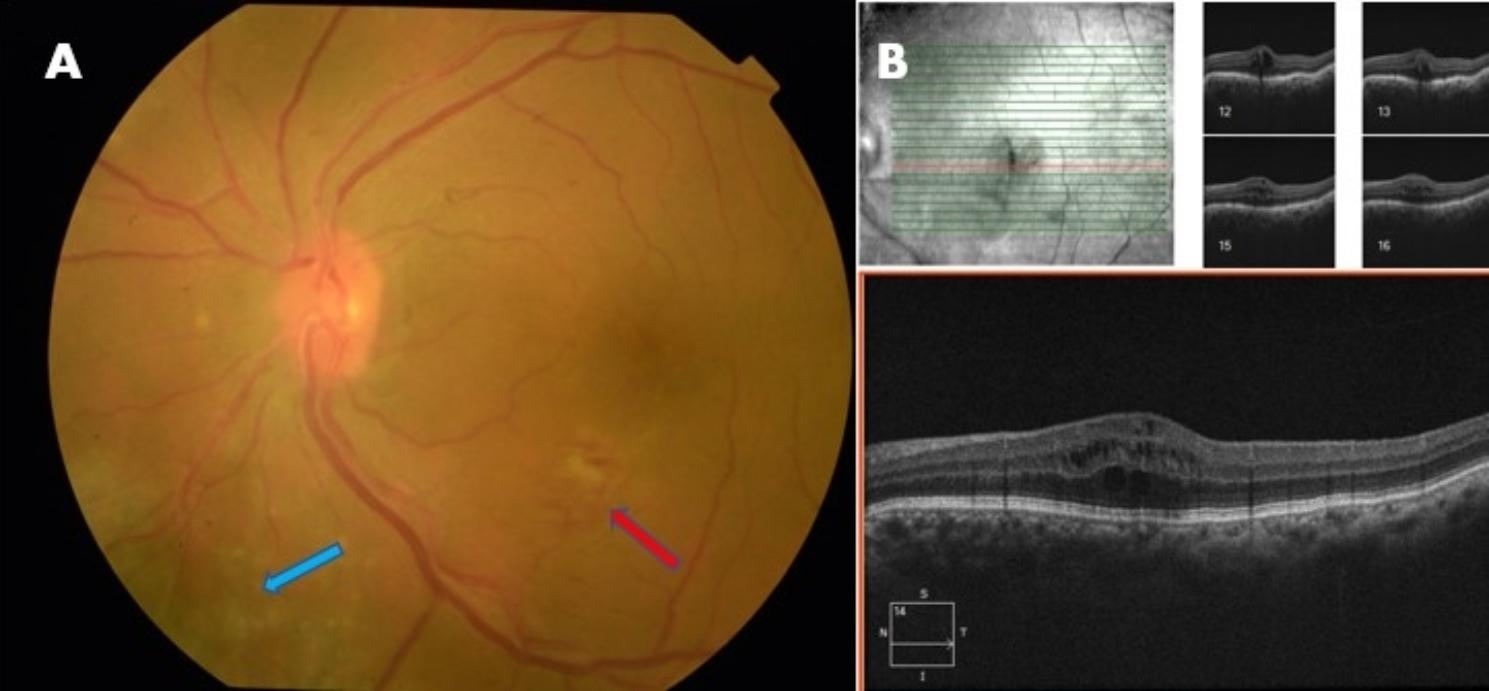
Color Fundus photo and OCT of the Left Eye. **A**. Fundus picture showing dull foveal reflex with macular edema with intra-retinal hemorrhages and cotton wool spots in the inferior macular region (red arrow) suggestive of macular BRVO, with yellowish flecks in the mid-peripheral region (blue arrow). **B**. OCT image shows elevated foveal contour with intra-retinal cystic spaces suggestive of Cystoid macular edema

A 56-year-old female presented to us with the best-corrected vision of 6/24 in both eyes with + 10.5 DS RE (Right eye) and + 10.75 DS LE (Left eye) respectively. Near vision was N6 in both eyes with + 2.25 DS. Ocular examination revealed a horizontal corneal diameter (HCD) of 10.5 mm in RE and 11 mm in LE with a shallow ACD (Van Herick’s grade 2) in both eyes. Both eyes had a patent peripheral iridotomy with RAPD (relative afferent pupillary defect) in the RE. On gonioscopy, both angles had 360-degree peripheral anterior synechiae with a clear lens. Her intraocular pressure (IOP) measured by applanation tonometry was 28 mm Hg in the RE and 14 mm Hg in the LE. She was on four anti-glaucoma medications (AGM) in RE (Travoprost, Pilocarpine, and Brimonidine-Timolol) and on Travoprost in LE. The retina had yellowish pigmentary flecks in the mid-peripheral zone in both eyes. RE had a medium size optic disc with a 0.9 cup: disc ratio with an Optic disc pit (ODP) in the superotemporal quadrant with accompanying subretinal fluid extending to the macular region (Fig. 1). LE had a medium-sized disc with 0.2 CDR, macular edema, and faint intraretinal hemorrhages in the inferior macular region (Fig. 2).

Her ocular biometric parameters have been described in Table 1.

**Table 1:** Ocular biometric parameters

Parameter	Right eye	Left eye
**AL**	16.78 mm (SD = 10 μm)	16.56 mm (SD = 10 μm)
**ACD**	1.85 mm (SD = 6 μm)	1.91 mm (SD = 5 μm)
**Lens thickness**	4.61 mm (SD = 12 μm)	4.60 mm (SD = 12 μm)
**Keratometry**	50.54 D	50.55 D
**Cylinder**	-0.48 D @ 87°	-0.84 D @ 47°
**White-to-white**	10.8 mm	11 mm
**IOL power (A constant- 118.2)- Formula- Hoffer Q**	+ 47 D (error- -0.14)	+ 49 D (error- -0.25D)

The patient did not give any history of consanguinity or night blindness. Systemically, she had hypertension controlled by medications. A diagnosis of bilateral NO with pigmentary retinopathy with RE optic disc pit associated with serous macular detachment with angle closure glaucoma (ACG) and LE angle closure and macular branch retinal venous occlusion (BRVO) secondary to hypertension was made. She was advised to undergo glaucoma filtering surgery in her right eye, however, she declined so AGMs were stepped up in the RE and an intravitreal anti-VEGF injection was planned for the LE.

## Discussion

An AL of 2 SD less than the mean for age, caused by a developmental ocular arrest is termed Microphthalmos. Depending on the measurements of the anterior and posterior segment, it is classified as Nanophthalmos (NO), Relative anterior microphthalmos (RAM), and Posterior Microphthalmos (PM).

A database by Rehlan et al. divided 38 patients with hyperopia > + 7D and AL < 20.5 mm, into two groups, NO and PM based on corneal diameter (11 mm as a benchmark). They reported PM to be the more common variant, with NO having a smaller corneal diameter (10.06 mm vs. 11.39 mm), shallower ACD (2.68 mm vs. 3.20 mm), thicker lens (4.77 mm vs. 3.93 mm), more incidence of angle closure disease (69% vs. 0%) and pigmentary retinal changes (38.6% vs. 8%). Macular folds were more common in the PM group. [6]

Our patient had HCD of 10.8 mm in the RE and 11 mm in the LE with shallow ACD in both eyes, ACG in the right eye, slightly increased lens thickness (normal lens thickness- 4.0-4.45 mm) [7], and pigmentary retinal changes in both eyes. All features other than the borderline corneal diameter prompted the diagnosis of bilateral NO.

Multiple case reports have noted a syndromic association of nanophthalmos, angle closure glaucoma, and pigmentary retinal degeneration, first described by Hermann et al. in 1958. [8] An additional finding of optic disc drusen and foveoschisis has been described by various authors. [5] The syndrome may be sporadic in occurrence or may follow an autosomal recessive or dominant trait. To date, five genes and 2 loci have been implicated. Out of these, mutation of the MFRP gene on Chr 11q23.3 has been found to cause the above-mentioned association. [5] This gene is expressed on both the retinal pigment epithelium and ciliary body and is required for both prenatal ocular growth and postnatal emmetropization.

Our case presented with the same spectrum of findings, the only difference being that instead of optic disc drusen, she had an optic disc pit. After a thorough literature search, we believe this is the first case to report an association of ODP with nanophthalmos and pigmentary retinopathy. Furthermore, she developed angle closure glaucoma only in the eye with the pit, despite having similar anterior chamber configuration in both eyes. We are not sure whether the pit developed secondary to the increased IOP, or glaucoma secondary to the pit (causing weakening of the lamina cribrosa).

ODP occurs as a result of herniation of dysplastic retinal tissue, which can lead to a defect in the lamina cribrosa and create an anomalous connection with the subarachnoid space. It is known to cause macular serous detachment/schisis in 25–75% of cases, with the origin of fluid being either the vitreous or the cerebrospinal space. In our case, the serous fluid causing the macular detachment would have to originate from the vitreous, as the high IOP would have prevented the cerebrospinal fluid (CSF) from accumulating. ODP is a rare anomaly with an estimated prevalence of 2 in 10,000, with no known genetic association. Yilmaz et al. have described a case with unilateral sectorial retinitis pigmentosa and ODP. [9] A similar case has been reported by Sheth et al. [10] However, none of these cases were associated with nanophthalmos, as in our case. Hence, we believe this to be a unique case, not previously reported in the literature.

There are certain shortcomings in our case report. The pedigree analysis would have helped in understanding the inheritance of her condition. The patient did not give a history of any ocular problems in her close blood relatives. Her parents were deceased, she had one elder brother and one son, who according to her had no ocular abnormality. Additionally, electrodiagnostic tests and screening for genetic mutations would have been helpful for genotype-phenotype correlation.

## Data Availability

The datasets used and/or analysed during the current study available from the corresponding author on reasonable request.

## Abbreviations

NO: Nanophthalmos
AL: Axial Length
SD: Standard Deviation
ACD: Anterior chamber depth
OPD: Out Patient Department
RE: Right eye
LE: Left eye
HCD: Horizontal corneal diameter
RAPD: Relative afferent pupillary defect
IOP: Intraocular pressure
ODP: Optic disc pit
CDR: Cup:Disc Ratio
IOL: Intraocular lens
ACG: Angle-closure glaucoma
AGM: Anti-Glaucoma Medications
VEGF: Vascular endothelial growth factor
RAM: Relative anterior microphthalmos
PM: Posterior microphthalmos
CSF: cerebrospinal fluid
